# Efficient Manipulation of Magnetic Domain Wall by Dual Spin‐Orbit Torque in Synthetic Antiferromagnets

**DOI:** 10.1002/advs.202514598

**Published:** 2025-10-17

**Authors:** Hiroto Masuda, Yuta Yamane, Takaaki Dohi, Takumi Yamazaki, Rajkumar Modak, Ken‐ichi Uchida, Jun'ichi Ieda, Mathias Kläui, Koki Takanashi, Takeshi Seki

**Affiliations:** ^1^ Institute for Materials Research Tohoku University Sendai 980‐8577 Japan; ^2^ Frontier Research Institute for Interdisciplinary Sciences Tohoku University Sendai 980‐8578 Japan; ^3^ Research Institute of Electrical Communication Tohoku University Sendai 980‐8577 Japan; ^4^ Institut für Physik Johannes Gutenberg‐Universität Mainz Staudingerweg 7 55128 Mainz Germany; ^5^ Research Center for Magnetic and Spintronic Materials National Institute for Materials Science Tsukuba 305‐0047 Japan; ^6^ Department of Advanced Materials Science, Graduate School of Frontier Sciences The University of Tokyo Kashiwa 277‐8561 Japan; ^7^ Advanced Science Research Center Japan Atomic Energy Agency Tokai 319‐1195 Japan; ^8^ Center for Science and Innovation in Spintronics Tohoku University Sendai 980‐8577 Japan; ^9^ International Center for Synchrotron Radiation Innovation Smart Tohoku University Sendai 980‐8577 Japan

**Keywords:** antisymmetric interlayer exchange coupling, magnetic domain wall motion, spin orbit torque, synthetic antiferromagnet

## Abstract

Current‐induced domain‐wall motion (CIDWM) in a synthetic antiferromagnet is a key phenomenon for developing potential high‐density‐packed magnetic domain‐wall memory with fast operation. Here, CIDWM is reported in the antiferromagnetically‐coupled two Co layers through the Ir interlayer sandwiched by the two Pt layers: Pt/Co/Ir/Co/Pt. The top and bottom Pt layers play a role for generating the spin current coming from the spin Hall effect, which gives rise to the dual spin‐orbit torque (SOT) acting on the perpendicular magnetizations of the Co layers. Although a simple argument would predict that SOTs from top and bottom Pt layers cancel each other out, the dual SOT nucleates a reversed magnetic domain and drives the CIDWM effectively at current density of the order of 10^11^ A m^−2^. This study also examines the effect of antisymmetric interlayer exchange coupling (AIEC) on CIDWM. A positive correlation between the magnitude of AIEC and the domain wall velocity is found, whereas the current density required for nucleating the reversed domain shows a negative correlation with the magnitude of AIEC. These facts suggest that the existence of AIEC improves the performance of CIDWM. The present results provide a new avenue to design SOT domain wall devices based on a synthetic antiferromagnet.

## Graphical abstract

Dual spin‐orbit torque coming from two Pt layers effectively drives the magnetic domain wall motion (DWM) in a synthetic antiferromagnet consisting of two Co ferromagnetic layers coupled with interlayer exchange coupling (IEC). In addition, the antisymmetric version of IEC leads to low current density for nucleating DW and fast velocity of DWM, improving the performance of magnetic DW devices.

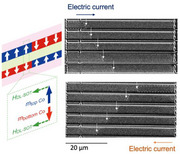

## 1 Introduction

Artificial magnetic systems with an antiferromagnetic alignment of the layers, which exploit the interlayer exchange coupling (IEC) in magnetic multilayers, are a treasure trove of functionalities for spintronics applications.^[^ ^1^, ^2^, ^3^, ^4^ ^]^ The ferromagnetic and nonmagnetic layers in a nanoscale heterostructure can each play a different role, making the total system highly functional. For example, two ferromagnetic layers such as Co separated by a nonmagnetic layer such as Cu, Ru, or Ir exhibit an antiferromagnetic alignment due to a long‐range IEC, and the magnitude of IEC, i.e., the strength of the antiferromagnetic coupling, can be tuned by varying the layer thicknesses.^[^ ^5^, ^6^, ^7^ ^]^ Such a controllability of antiferromagnetic properties is a feature that is not found in bulk antiferromagnets. A recent research trend in spintronics, aiming to utilize the characteristics of antiferromagnetic materials such as the low magnetic susceptibility, lack of the magnetic stray field, and high antiferromagnetic resonance frequencies, is called antiferromagnetic spintronics.^[^ ^8^ ^]^ The antiferromagnetic structures artificially formed in magnetic multilayers are a suitable platform for systematic study of the antiferromagnetic spintronics thanks to the controllability of their antiferromagnetic properties as mentioned above. Also, perpendicular magnetic anisotropy can be induced at the interfaces in a multilayer, which realizes a perpendicularly‐magnetized synthetic antiferromagnet. In addition, the broken inversion symmetry at the interfaces gives rise to the interfacial Dzyaloshinskii‐Moriya interaction, leading to formation of topological magnetic structures such as a skyrmion.^[^ ^9^ ^]^ Particularly, the antiferromagnetic skyrmion observed in an IEC multilayer ^[^ ^10^, ^11^, ^12^, ^13^ ^]^ is of interest due to the drastically changed spin dynamics resulting from the antiferromagnetic coupling of the layers. The synthetic antiferromagnets are also useful for developing high‐performance spintronic devices,^[^ ^14^, ^15^, ^16^, ^17^ ^]^ particularly based on domain walls (DWs). A previous study demonstrated a fast current‐induced domain‐wall motion (CIDWM) in a synthetic antiferromagnet Pt/(Co/Ni)/Ru/(Co/Ni).^[^ ^17^ ^]^ They exploited the exchange coupling torque as well as the spin orbit torque (SOT) induced by the spin Hall effect (SHE) in Pt.^[^ ^18^ ^]^

Our previous study ^[^ ^19^ ^]^ realized a current‐induced magnetization switching in Pt/Co/Ir/Co/Pt multilayers. The two Co layers were perpendicularly magnetized and either antiferromagnetically or ferromagnetically coupled through the Ir interlayer, depending on the Ir layer thickness. The dual SOT originating from the top and bottom Pt layers acts on the magnetizations in the two Co layers. Through magnetic domain observation of the SOT switching, we found that the antiferromagnetic alignment is favorable for stable and efficient SOT‐switching operations.

Apart from the SOT‐related phenomena, an antisymmetric IEC (AIEC) between the two ferromagnetic layers was observed in Pt/Co/Ir/Co/Pt with wedge‐shaped layers.^[^ ^20^ ^]^ The AIEC is induced due to the broken inversion symmetry along the in‐plane direction, which was first theoretically predicted,^[^ ^21^ ^]^ then experimentally observed.^[^ ^22^, ^23^ ^]^ After the early work confirming the existence of AIEC,^[^ ^22^, ^23^, ^24^ ^]^ the following studies revealed the characteristics of AIEC,^[^ ^20^, ^25^, ^26^, ^27^, ^28^, ^29^, ^30^, ^31^, ^32^, ^33^, ^34^, ^35^ ^]^ e.g., the relation between IEC and AIEC.^[^ ^20^, ^25^, ^26^ ^]^ The conventional, symmetric IEC energy is expressed as *J*_AF_(**m**_A_ · **m**_B_), where *J*_AF_ denotes the antiferromagnetic coupling strength and **m**_A_ and **m**_B_ are the unit vectors representing the magnetizations in the two ferromagnetic layers, A and B. On the other hand, the AIEC energy is expressed as **D**_AIEC_ · (**m**_A_ × **m**_B_), where **D**_AIEC_ is the AIEC vector determined by system symmetry. With the film normal direction along the *z* direction and the inversion symmetry breaking along the *y* direction, **D**_AIEC_ appears along the *x* direction. The AIEC prefers a noncolinear alignment of **m**_A_ and **m**_B_, leading to chiral magnetic configurations that can be exploited to realize artificial 3D topological magnetic structures.^[^ ^36^ ^]^ Microscopic mechanisms of AIEC and its applications to the spintronic devices are therefore attracting considerable attention. Several experimental studies demonstrated that the AIEC allows the field‐free SOT switching thanks to the in‐plane effective field originating from the AIEC field.^[^ ^28^, ^29^, ^30^, ^31^ ^]^ However, there is no experimental elucidation how the AIEC field affects the CIDWM. Also, the behavior of DW under the dual SOT application is not a trivial issue because a simple picture of dual SOT gives the cancellation between the SOTs from top and bottom spin Hall layers. Considering the complex layer stacking incorporated into the current spintronics devices, the understanding of the mechanism and process of the SOTs coming from multiple layers is remarkably important from the viewpoint of not only an academic interest but also an efficient device operation.

This paper presents a combined experimental and theoretical study on CIDWM in a perpendicularly‐magnetized synthetic antiferromagnet Pt/Co/Ir/Co/Pt with AIEC. We find that the dual SOT can effectively drive DWM in the presence of in‐plane field, despite the opposite polarization of the spin current injected from the top and bottom Pt layers. We find that the AIEC can reduce the current density required for DW nucleation and increase the DW velocity. The experimental observations are consistent with our numerical simulation. The present results provide a new direction to design DW devices using a synthetic antiferromagnet.

## 2 Experimental Results

**Figure 1:**
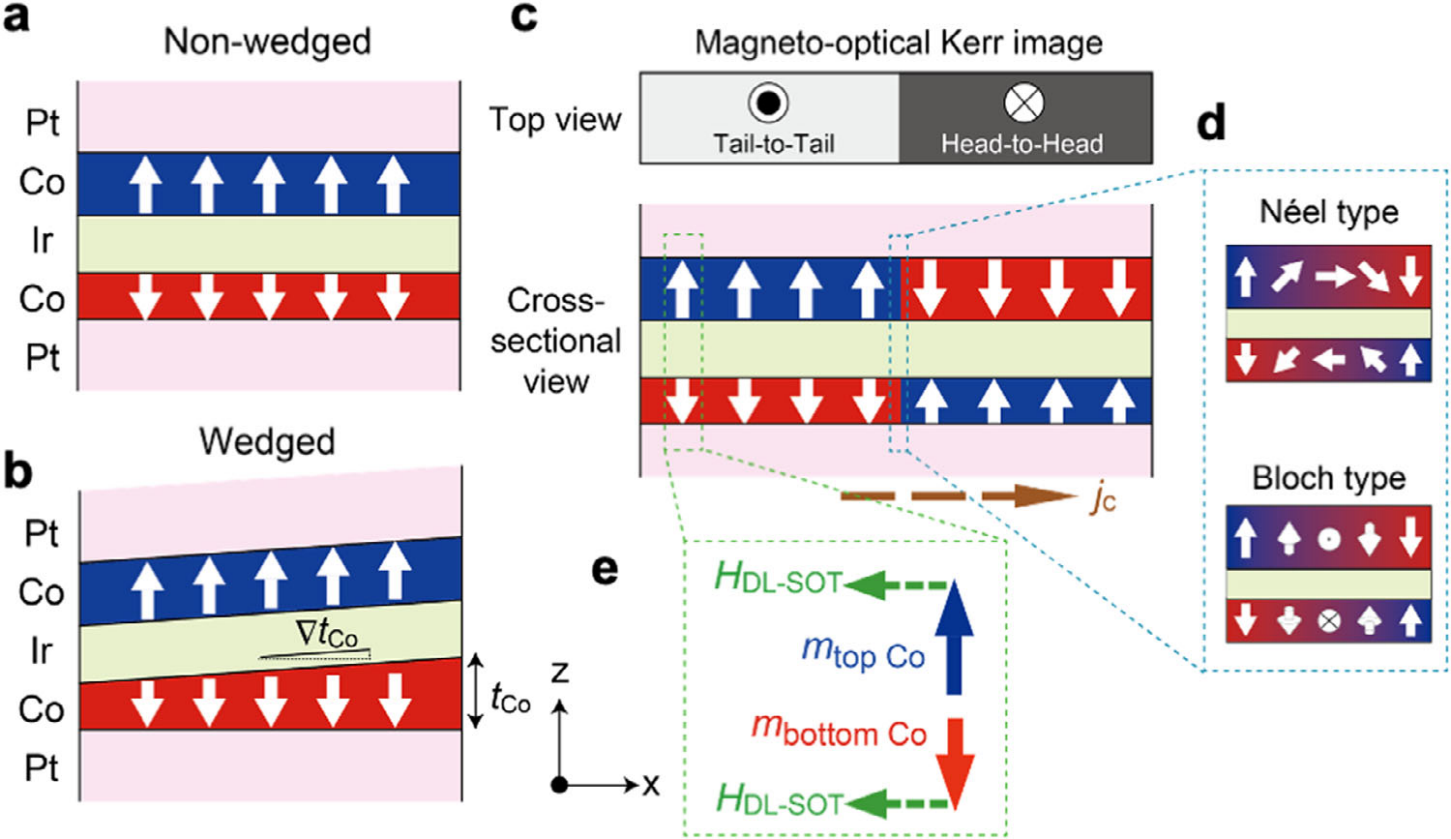
Schematic illustration of stacking structures together with the magnetization configuration of two Co layers for a) non‐wedged sample and b) wedged sample. c) Expected magneto‐optical Kerr image for a sample with tail‐to‐tail (light gray) and head‐to‐head (dark gray) magnetic domains, where the tail‐to‐tail domain possesses an upward net magnetization while the head‐to‐head domain possesses a downward net magnetization. d) Illustration of magnetic structures inside Néel‐type and Bloch‐type domain walls. e) Effective field of damping‐like (DL) spin‐orbit torque (SOT) *H*_DL‐SOT_ acting on the top and bottom Co magnetic moments.

The following layer stackings were deposited on thermally oxidized Si substrates using a magnetron sputtering at room temperature: Si‐O Subs.//Ta (2)/Pt (3)/Co (0.65 or *t*_Co_)/Ir (1.3)/Co (0.9)/Pt (3)/Ta (1) (thickness in nanometer). **Figure** **1**a illustrates the central layer stacking together with the magnetization configuration of two Co layers. The 1.3 nm‐thick Ir layer leads to the antiferromagnetic IEC, and the 1.3 nm thickness is the second peak position of oscillatory behavior of IEC strength as a function of the Ir interlayer thickness.^[^ ^19^ ^]^ Thanks to the interfaces with the Pt layers, the top and bottom Co layers are perpendicularly magnetized. In order to intentionally break the in‐plane spatial inversion, the bottom Co layers were designed to have a wedge shape (Figure 1b). In our previous study,^[^ ^20^ ^]^ the wedged Co layer was effective to induce the AIEC. As discussed later, although there are several sources for the AIEC except the wedge shape, implying that the AIEC field is determined by multiple contributions as we reported in another paper,^[^ ^35^ ^]^ we consider that the wedge shape becomes one of major sources breaking the in‐plane spatial inversion. For the Co‐wedged samples, *t*_Co_ denotes the thickness of wedged bottom Co layer, and the ranges of *t*_Co_ were set to be 0.6 ≤ *t*_Co_ ≤ 1.1 nm, 0.4 ≤ *t*_Co_ ≤ 1.4 nm, and 0.3 ≤ *t*_Co_ ≤ 1.8 nm, which correspond to the thickness gradient ∇*t*_Co_ of 0.6 × 10^−7^, 1.1 × 10^−7^, and 1.7 × 10^−7^, respectively. For the thin film without the wedged layer, the Co layer thickness was fixed at 0.65 nm, which is called non‐wedged sample in this study. It is noted that out‐of‐plane direction is the easy magnetization direction for all *t*_Co_ in this study, which was confirmed by measuring the polar magneto‐optical Kerr effect loops (Figure S1, Supporting Information).

In order to observe magnetic domain structures and characterize their domain wall motion, magneto‐optical Kerr effect imaging was employed in this study. As depicted in Figure 1c, the observed domain image mainly reflects net magnetization, which is the same direction as the top Co magnetization because the top Co layer is thicker than the bottom Co layer. The possible magnetic structures in the domain wall, that is, Néel‐type and Bloch‐type domain walls are also illustrated in Figure 1d. Since the dual‐SOT acts on the magnetic moments of Co layers under the electric current application, the effective magnetic field of damping‐like (DL) SOT *H*_DL‐SOT_ is also depicted in Figure 1e.

**Figure 2:**
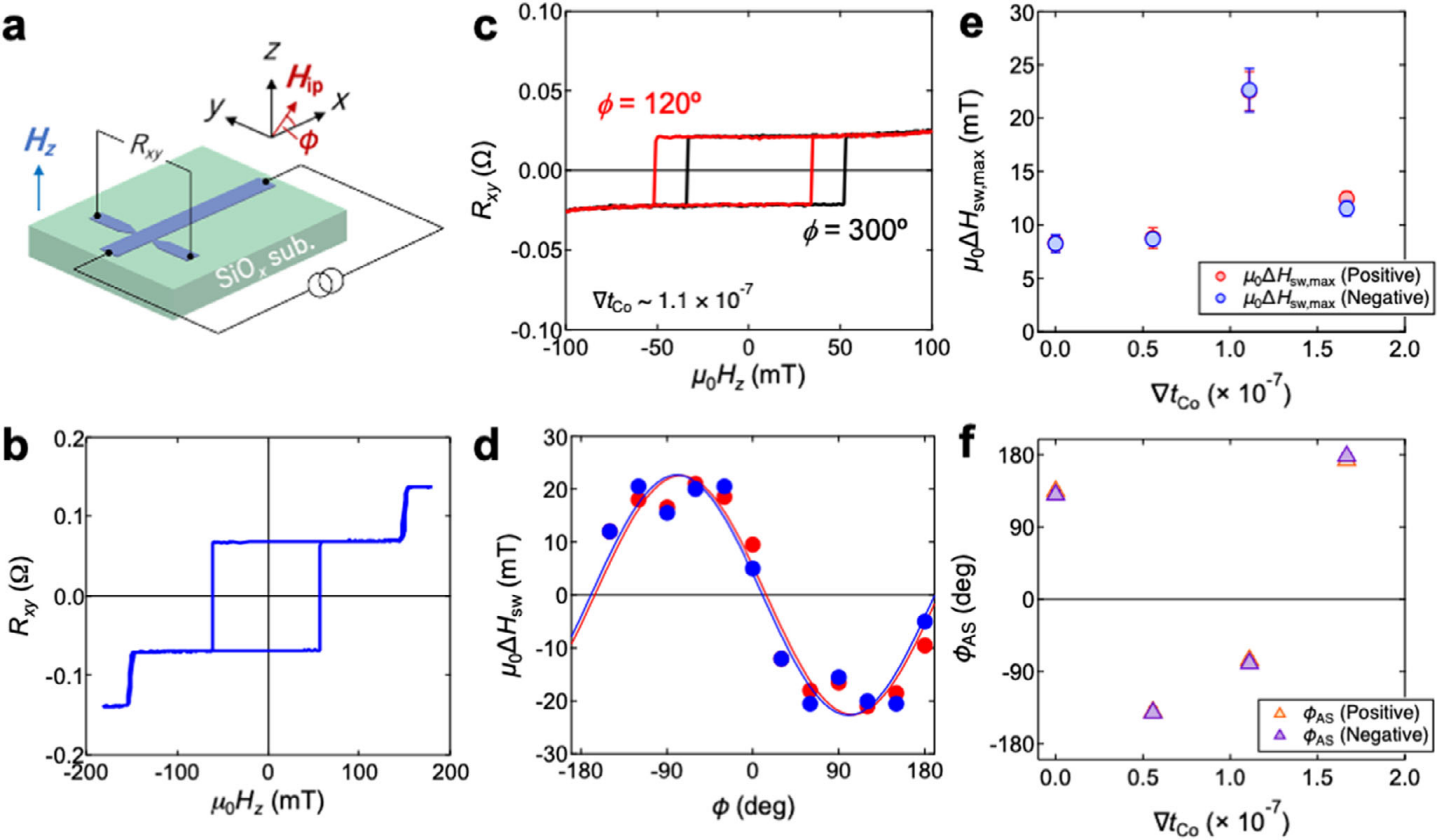
Characterization of symmetric interlayer exchange coupling (IEC) and antisymmetric IEC (AIEC). a) Schematic illustration of a Hall‐bar device with the coordinate, where the in‐plane field angle of *H*_ip_ is defined as the angle *ϕ*_AS_ from the *x*‐axis. b) Transverse resistance *R_xy_* as a function of perpendicular magnetic field *H*_*z*_ for the device of the non‐wedged sample. c) *R_xy_* curves for the device with ∇*t*_Co_ = 1.1 × 10^−7^, where *μ*_0_ *H*_ip_ = 50 mT was applied at *ϕ*_AS_ = 120° (red curve) and 300° (black curve). d) *ϕ*_AS_ dependence of the unidirectional shift in switching field Δ*H*_sw_ for the device with ∇*t*_Co_ = 1.1 × 10^−7^ at *μ*_0_ *H*_ip_ = 50 mT. The red and blue circles denote Δ*H*_sw_ obtained from *H*_sw_ in the positive and negative field regions, respectively. The solid curves are the fitting results with cosine function. e) *μ*_0_Δ*H*_sw,max_ and f) *ϕ*_AS_ as a function of ∇*t*_Co_, where the red and blue marks denote the results obtained from *H*_sw_ in the positive and negative field regions, respectively.* μ*_0_Δ*H*_sw,max_ and *ϕ*_AS_ are the fitting parameters of *μ*_0_Δ*H*_sw_ = *μ*_0_Δ*H*_sw,max_ cos(*ϕ* – *ϕ*_AS_), which was used in d).

The thin films were patterned into a Hall‐bar‐shape (**Figure** **2**a) with a 5 µm width‐channel. The direction of wedge shape in the bottom Co layer is along to the *x‐*axis of cartesian coordinates as shown in Figure 1b. Figure 2b displays the transverse resistance *R_xy_* as a function of perpendicular magnetic field *H*_*z*_ for the device of the non‐wedged sample, where *R_xy_* mainly comes from the anomalous Hall effect proportional to the perpendicular component of net magnetization of two Co layers. The two Co magnetizations are antiferromagnetically aligned at low *H*_*z*_, and those are saturated ferromagnetically as *H*_*z*_ is increased to 200 mT. Similar to the non‐wedged sample, all the samples exhibit the two Co magnetizations coupled antiferromagnetically each other due to the symmetric IEC.

In order to evaluate the magnitude of AIEC, the *R_xy_*–*H*_z_ curves were measured with the additional in‐plane magnetic field *H*_ip_. The in‐plane field angle of *H*_ip_ is defined as the angle *ϕ*_AS_ from the *x*‐axis (see Figure 2a). Figure 2c shows the *R_xy_*–*H*_z_ curves for the device with ∇*t*_Co_ = 1.1 × 10^−7^, where *μ*_0_ *H*_ip_ = 50 mT was applied at *ϕ*_AS_ = 120° (red curve) and 300° (black curve). In the presence of AIEC, the application of *H*_ip_ leads to the asymmetry in the up‐to‐down (UD) and down‐to‐up (DU) switching fields for the net magnetization due to the preferred magnetic chirality dictated by the AIEC. As a result, the unidirectional shift of *R_xy_* – *H*_z_ curve appears by applying *H*_ip_. The magnitude of unidirectional shift, related to the AIEC, is represented by Δ*H*_sw_, which is obtained from the difference in the switching fields *H*_sw_ with *H*_ip_ at *ϕ*_AS_ and *ϕ*_AS_+ π. The value of Δ*H*_sw_ is an indicator of the magnitude of AIEC. Figure 2d plots the* ϕ*_AS_ dependence of *μ*_0_Δ*H*_sw_ for the device with ∇*t*_Co_ = 1.1 × 10^−7^ at *μ*_0_ *H*_ip_ = 50 mT. The experimental data are fitted by *μ*_0_Δ*H*_sw_ = *μ*_0_Δ*H*_sw,max_ cos(*ϕ*_AS_ – *ϕ*_AS_).* μ*_0_Δ*H*_sw,max_ represents the magnitude of AIEC, and *ϕ*_AS_ is the direction of AIEC, which corresponds to the in‐plane direction orthogonal to **D**_AIEC_. *μ*_0_ is the permeability of the vacuum. Figure 2e,f summarize *μ*_0_Δ*H*_sw,max_ and *ϕ*_AS_, respectively, as a function of ∇*t*_Co_. Here, ∇*t*_Co_ = 0 means the non‐wedged sample. Originally we anticipated that the increase of ∇*t*_Co_ simply increases the magnitude of AIEC (Δ*H*_sw,max_) and the direction AIEC (*ϕ*_AS_) is aligned along the wedge direction. As can be seen, however, the device with ∇*t*_Co_ = 1.1 × 10^−7^ exhibits the largest Δ*H*_sw,max_ among the present devices. Even the non‐wedged sample shows non‐negligible Δ*H*_sw,max_. In addition, the *ϕ*_AS_ deviates from the expectation, that is, the *x*‐axis (*ϕ*_AS_ = 0° or 180°). These facts suggest that apart from the wedge shape in the bottom Co layer there exist other sources providing the AIEC, e.g., the thickness inhomogeneity and/or the growth‐induced magnetic anisotropy. Those other contributions were suggested also in previous studies.^[^ ^19^, ^23^, ^35^ ^]^ Thus, the direction and magnitude of AIEC cannot precisely be controlled by changing the thickness gradient in this study. Nevertheless, by introducing the wedge‐shaped Co layers with different thickness gradients, a series of samples exhibiting different AIEC fields allow us to examine the influence of AIEC on the CIDWM.

**Figure 3:**
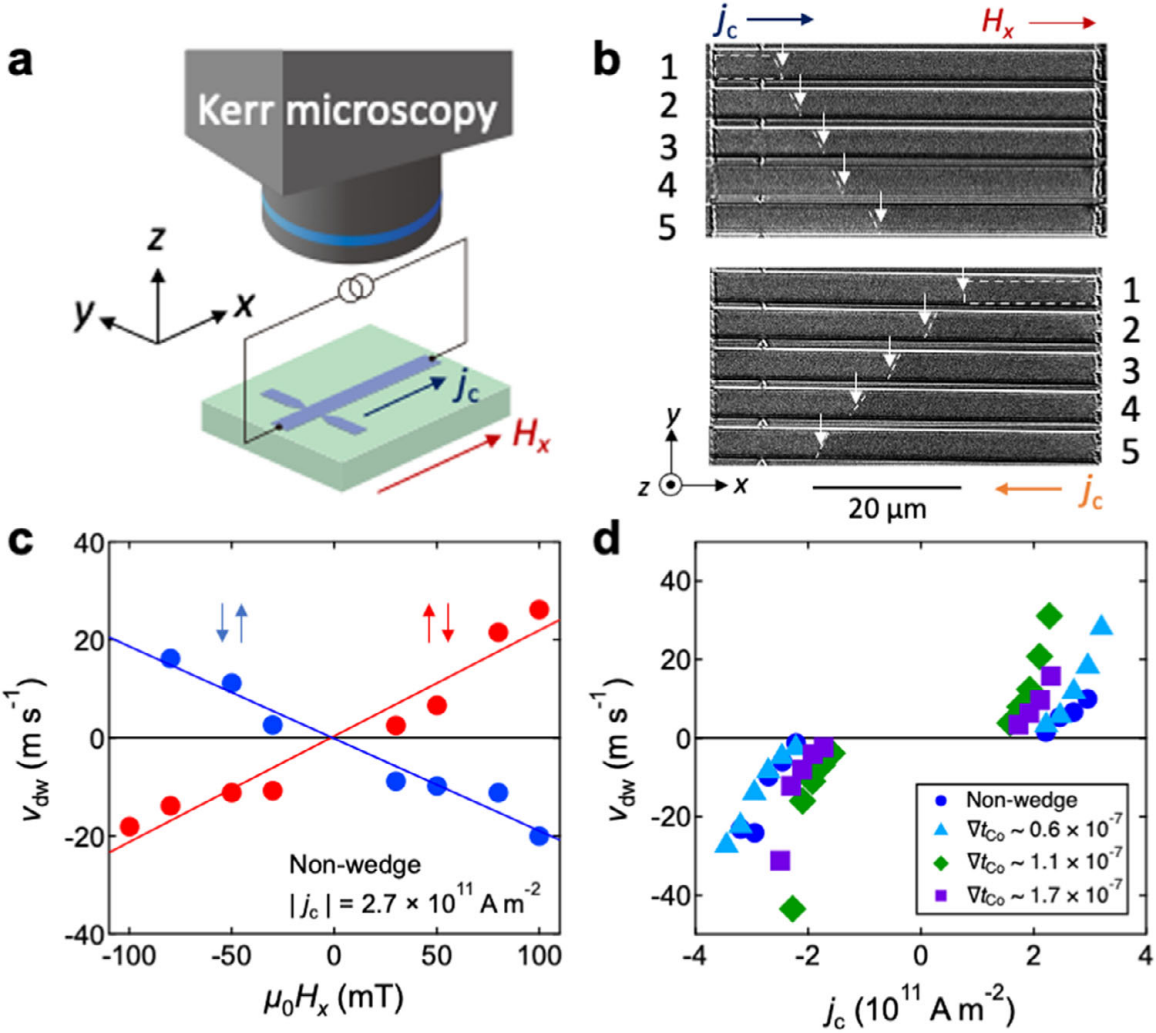
a) Experimental setup for the current‐induced domain wall motion (CIDWM) observation using the magneto‐optical Kerr microscope. b) Magneto‐optical Kerr images of CIDWM for the non‐wedged device at *μ*_0_ *H_x_* = 50 mT. Top (bottom) panel shows the contrast change after applying *j*_c_ = 2.7 × 10^11^ A m^−2^ with a pulse width of 500 ns in the +*x* (−*x*) direction. c) Velocity of DWM *v*_dw_ as a function of in‐plane magnetic field *H_x_* for the non‐wedged device under the application of *j*_c_ = 2.7 × 10^11^ A m^−2^. Red (blue) circles represent the data obtained for the device with the initial state of head‐to‐head (tail‐to‐tail) configuration. d) *v*_dw_ as a function of *j*_c_ for the non‐wedged device and the devices with ∇*t*_Co_ = 0.6 × 10^−7^, 1.1 × 10^−7^, and 1.7 × 10^−7^, in which *μ*_0_ *H_x_* = 50 mT was applied.

**Figure** **3**a is the setup for the CIDWM observation using the magneto‐optical Kerr microscope. The representative results are given in Figure 3b, which were observed for the non‐wedged device. One sees that there are two gray contrasts: dark gray and light gray. The dark gray and light gray regions correspond to the magnetic domains with the head‐to‐head and tail‐to‐tail configurations, respectively, of magnetic moments between the top and bottom Co layers as depicted in Figure 1c. For the present CIDWM experiment, the reversed magnetic domain was nucleated at a certain position in the wire when the current pulse with a certain current density *j*_c_ was applied. Although this reversed domain nucleation procedure is not a precisely‐controlled manner, we could successfully nucleate the reversed domain in almost the middle of wire. The domain nucleation was attributable to the switching due to the dual SOT. The positions of DWs are denoted by the white arrows. The top (bottom) panel shows the contrast change after the application of *j*_c_ = 2.7 × 10^11^ A m^−2^ and with a pulse width of 500 ns in the +*x* (−*x*) direction, where *μ*_0_ *H_x_* = 50 mT was applied. The DW moves along the electric current direction (opposite to the direction of electron flow) with increasing the number of current pulse application. This tendency can be explained with the scenario of DWM induced by SOT.^[^ ^37^, ^38^, ^39^ ^]^ The spin Hall effect in the Pt and/or Ir layer generates the spin current interacting with the Co magnetizations. According to the SOT switching experiment reported previously,^[^ ^19^ ^]^ the major source of SOT is the spin Hall effect in the Pt layers while the spin Hall effect in the Ir interlayer is not significant. Thus, the SOT coming from the top and bottom Pt layers act on the top and bottom Co magnetizations individually.

The velocity of DWM *v*_dw_ is evaluated from the distance of DW movement and the duration of current pulse. Figure 3c plots *v*_dw_ as a function of *H_x_* for the non‐wedged device under the application of *j*_c_ = 2.7 × 10^11^ A m^−2^. The red (blue) circles represent the data obtained for the device with the initial state of head‐to‐head (tail‐to‐tail) configuration. Considering the experimental situation depicted in Figure 1, the tail‐to‐tail region having the upward net magnetization (↑) is created in the head‐to‐head region having the downward net magnetization (↓) and the region with ↑ is expanded by the CIDWM. Thus, the experiment starting from the head‐to‐head configuration corresponds to the CIDWM for the up‐down (↑↓) DW while the tail‐to‐tail initial configuration leads to the down‐up (↓↑) DW. Both cases exhibit the linear changes in *v*_dw_ against *H_x_*, but show the opposite signs in the slopes. As can be seen, *v*_dw_ is almost equal to zero at *μ*_0_ *H_x_* = 0 mT. These experimental facts indicate that in line with previous findings the contribution of spin transfer torque within the Co layers is negligible while the SOT is the dominant source of CIDWM. The detailed discussion will be given later. Figure 3d summarizes *v*_dw_ as a function of *j*_c_ for the non‐wedged device and the devices with ∇*t*_Co_ = 0.6 × 10^−7^, 1.1 × 10^−7^, and 1.7 × 10^−7^, in which *μ*_0_ *H_x_* = 50 mT was applied. After the nucleation of reversed domain at a certain *j*_c_, which is defined as the nucleation current density *j*_nucl_, *v*_dw_ nonlinearly increases with increasing *j*_c_ for all the devices.

**Figure 4:**
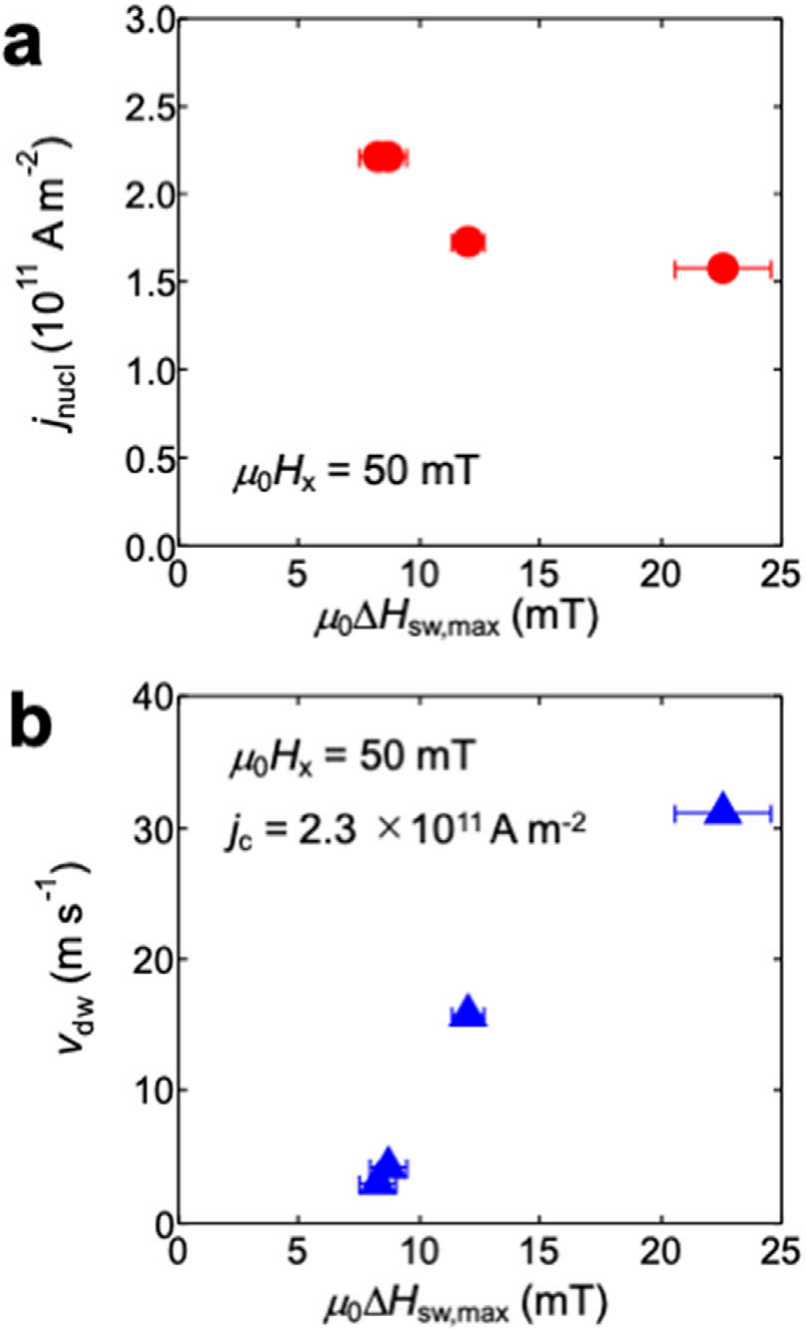
Effect of AIEC on nucleation current density *j*_nucl_ and *v*_dw_ for the CIDWM. a) *j*_nucl_ obtained at *μ*_0_ *H_x_* = 50 mT as a function of Δ*H*_sw,max_. b) *v*_dw_ as a function of Δ*H*_sw,max_, where *v*_dw_ was evaluated under the application of *j*_c_ = 2.3 × 10^11^ A m^−2^ and *μ*_0_ *H_x_* = 50 mT.

To discuss the effect of AIEC on the CIDWM, we plots the *j*_nucl_ obtained at *μ*_0_ *H_x_* = 50 mT as a function of Δ*H*_sw,max_ in **Figure** **4**a. One can see that there is a negative correlation between *j*_nucl_ and Δ*H*_sw,max_. Namely, the AIEC field promotes the nucleation of reversed domain under the dual SOT application. On the other hand, *v*_dw_ clearly increases with Δ*H*_sw,max_ as shown in Figure 4b, where *v*_dw_ was evaluated under the application of *j*_c_ = 2.7 × 10^11^ A m^−2^ and *μ*_0_ *H_x_* = 50 mT. At this condition, the fastest *v*_dw_ was obtained to be 31 m s^−1^ for the device with ∇*t*_Co_ = 1.1 × 10^−7^, which is one order of magnitude larger than the *v*_dw_ for the non‐wedged device. The positive correlation between *v*_dw_ and Δ*H*_sw,max_ means that the AIEC field plays a role for the efficient CIDWM. According to the previous works,^[^ ^27^, ^35^ ^]^ the oblique sputter‐deposition or the selection of nonmagnetic interlayer with large spin‐orbit coupling can enhance the magnitude of AIEC, leading to the further improvement of CIDWM efficiency. One may be wondering how the degree of compensation between top and bottom Co magnetizations affects *v*_dw_. A previous work reported that the closely compensated state leads to the fast *v*_dw_.^[^ ^17^ ^]^ Although the wedged bottom Co layers give a spatial change in the degree of compensation, we do not observe any relation between *v*_dw_ and ∇*t*_Co_ in the present system. In addition, one may think why even the *y*‐directional AIEC field positively affects DW velocity. We consider that the present case is still far from the ideal CIDWM showing the linear correlation between *v*_dw_ and *j*_c_. Rather the nucleation energy and the pinning potential for DW determine the process for CIDWM, which may be affected by the AIEC field. This is a possible explanation for the fact that the AIEC field positively affects DW velocity regardless of *ϕ*_AS_.

## 3 Theoretical Calculation and Discussion

In order to understand the CIDWM by dual SOT, we carry out micromagnetic simulations in a synthetic antiferromagnetic nanowire with a custom‐developed code. Here, we focus to elucidate the following two points: i) How the dual SOT can drive the DWM despite that a simple argument predicts that the spin injections from the top and bottom Pt layers with the opposite spin polarizations lead to a net zero effect on the magnetization dynamics. ii) How the AIEC affects the CIDWM.

We model our synthetic antiferromagnet by the following surface magnetic energy density (*U*),

(1) $$\begin{array}{ccc} U & = & J_{\mathrm{AF}}m_{A}\cdot m_{B}+D_{\mathrm{AIEC}}\cdot \left(m_{A}\times m_{B}\right)+A\sum_{i=x,y,z}\left[t_{A}{\left(\partial_{i}m_{A}\right)}^{2}\right.\\ & & +\left.t_{B}{\left(\partial_{i}m_{B}\right)}^{2}\right]-K\left[t_{A}{\left(m_{A,z}\right)}^{2}+t_{B}{\left(m_{B,z}\right)}^{2}\right]\\ & & -\;\mu_{0}M_{s}H\cdot \left(t_{A}m_{A}+t_{B}m_{B}\right) \end{array}$$

where *A* is the exchange stiffness, *K* is the perpendicular magnetic anisotropy constant, *M*_s_ is the saturation magnetization, **H** is the external magnetic field, and *t*_A(B)_ is the thickness of the layer A (B). The dynamics of **m** *_µ_* (*µ* = A, B) are described by the coupled Landau‐Lifshitz‐Gilbert (LLG) equations

(2) $$\frac{\partial m_{\mu }}{\partial t}=-\gamma m_{\mu }\times H_{\mu }+\alpha m_{\mu }\times \frac{\partial m_{\mu }}{\partial t}-\gamma m_{\mu }\times \left(m_{\mu }\times H_{\mu }^{\mathrm{SOT}}\right)$$

where *γ* is the gyromagnetic ratio and *α* is the damping constant. The effective magnetic fields **H** *_µ_* are defined by **H**_*μ*_ = − (*μ*_0_ *M_s_t*_*μ*_)^−1^(δ*U*/δ**m**_*μ*_). The last term in Equation (2) describes the dual SOT, which is characterized by the effective fields

(3) $$H_{\mu }^{\mathrm{SOT}}=\pm \frac{\hbar \theta_{\mathrm{SH}}}{2e\mu_{0}M_{s}t_{\mu }}j_{c}y$$

with the upper (lower) sign corresponding to *µ* = A (B) and *θ*_SH_ the effective spin Hall angle.

Under the surface energy density with **H** = 0, the system can accommodate a stable DW in equilibrium.^[^ ^40^ ^]^ We prepare a Bloch DW as the initial state, i.e., the magnetizations rotate in the *yz* plane in the DW region, and then examine the dynamics of the DW in the presence of current and magnetic field. We choose a Bloch DW since both the dual SOT and *H_x_* act to stabilize a Bloch structure: The dual SOT with Equation (2) simply tries to align **m**_A_ and **m**_B_ along + **y** and − **y**, respectively, while a Bloch structure can also reduce the Zeeman energy by allowing for the magnetizations to slightly cant toward the *x* direction. We assume |**D**_AIEC_| to be sufficiently small that the influence of the AIEC on the DW structure can be ignored. The parameter set used is: *M*_s_ = 1.1 × 10^6^ A m^−1^,  *α* = 0.06,  *J*_AF_ = 0.5 × 10^−3^ J m^−2^, *A* = 1 × 10^−11^ J m^−1^, *K* = 2 × 10^5^ J m^−3^,  *t*_A_ = 0.9 nm, and *t*_B_ = 0.65 nm. The values of *M*_s_, *K* and *J*_AF_ are taken from experimental data on our Co/Ir/Co system, ^[^ ^19^ ^]^ *α* from reported values for similar systems, ^[^ ^41^ ^]^ and *A* from a typical value for ferromagnets.^[^ ^42^ ^]^ We introduce the in‐plane angle *θ*_D_(= *ϕ*_AS_ − π/2 ) of the AIEC as **D**_AIEC_ = |**D**_AIEC_| (cos *θ*_D_, sin *θ*_D_, 0).

**Figure 5:**
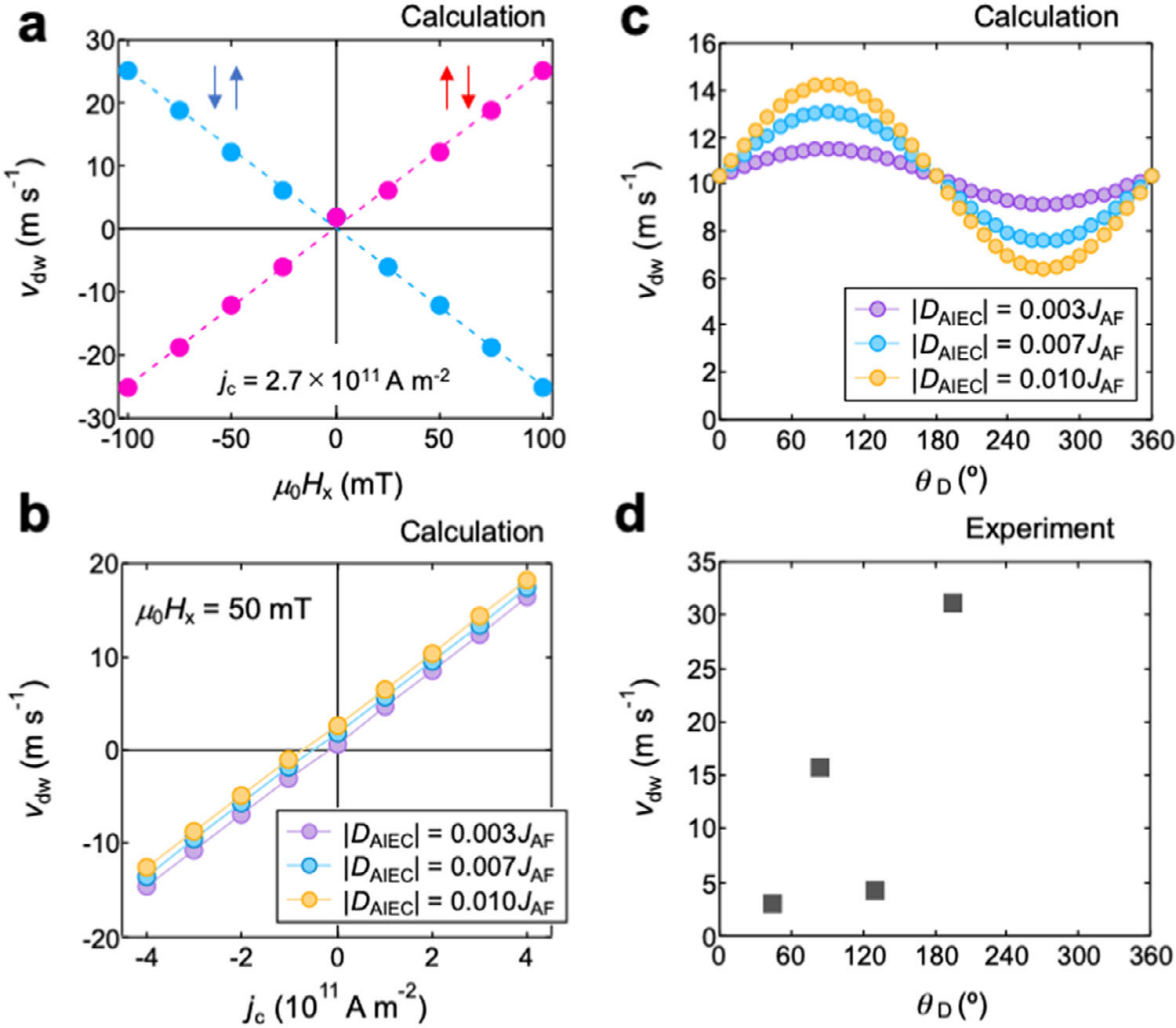
a) Calculated *v*_dw_ as a function of *H_x_* under the application of *j*_c_ = 2.7 × 10^11^ A m^−2^. The magenta (cyan) marks represent the calculated values for the up‐down DW (down‐up DW). b) Calculated *v*_dw_ as a function of *j*_c_ with changing *D*_AIEC_, where *μ*_0_ *H_x_* was set to be 50 mT. c) Calculated *v*_dw_ as a function of the in‐plane angle of *D*_AIEC_, *θ*_D_ under the application of *j*_c_ = 2.7 × 10^11^ A m^−2^, and *μ*_0_ *H_x_* = 50 mT. d) Experimental *v*_dw_ as a function of *θ*_D_ under the application of *j*_c_ = 2.3 × 10^11^ A m^−2^ and *μ*_0_ *H_x_* = 50 mT.

**Figure** **5**a plots the calculated *v*_dw_ as a function of *H_x_* under the application of *j*_c_ = 2.7 × 10^11^ A m^−2^, where the magenta (cyan) symbols correspond to the results for the up‐down (down‐up) DW. Here, the AIEC is set to |**D**_AIEC_| = 0.007*J*_AF_ and *θ*_D_ =  45°. The calculation reproduces quantitatively well the major experimental features observed in Figure 3c: the linear dependence of *v*_dw_ on *H_x_*, the sign reversal of *v*_dw_ between the up‐down and down‐up DW configurations, and no efficient CIDWM without *H_x_*.

Figure 5b shows the *j*_c_ dependence of the calculated *v*_dw_ with several different |**D**_AIEC_|, where *θ*_D_ = 45° and *μ*_0_ *H_x_* = 50 mT. *v*_dw_ exhibits linear dependence on *j*_c_, and shifts vertically as |**D**_AIEC_| varies. The nonzero *v*_dw_ at *j*_c_ = 0 is due to the combined effect of the AIEC and *H_x_*,^[^ ^40^ ^]^ as discussed in more detail below. The result in Figure 5b confirms that the dual SOT is mainly responsible for the observed DWM. The calculated CIDWM shows zero threshold current density, in contrast to the experiment (Figure 3d). The threshold in *j*_c_ for CIDWM may be attributed to the potential barrier for nucleating the reversed domains as well as pinning potentials due to impurities, which are not taken into account in the presented calculation.

Displayed in Figure 5c is the *θ*_D_ dependence of *v*_dw_, with *j*_c_ = 2.7 × 10^11^ Am^−2^ and *μ*_0_ *H_x_* = 50 mT. As elaborated on shortly, this *θ*_D_ dependence originates from the fact that only the *y* component of **D**_AIEC_ gives a major contribution to *v*_dw_. Figure 5d plots the experimentally‐observed *v*_dw_ as a function of *θ*_D_. It is difficult to make a direct and systematic comparison between the experiment and calculation for technical reasons. The number of experimental data points is limited, because only a small number of devices with the desired *t*_Co_ can be obtained from the wedged samples. In addition, precise control of the direction and magnitude of the AIEC field remains difficult in experiments. Despite these limitations, however, our results clearly demonstrate that the DW velocity can be tuned by engineering the AIEC. A more direct comparison between the experimental and numerical results is given in Figure S2 (Supporting Information).

Here, we consider the physical mechanism underlying the DWM driven by the dual SOT. For the sake of simplicity, we assume the antiferromagnetic limit where *t*_A_ = *t*_B_ and *J*_AF_ is by far the most dominant energy scale in *U*. Starting from the coupled LLG equations (2), we rewrite them in terms of the Néel vector $n=\frac{m_{A}-m_{B}}{2}$ and the canting moment $m\;=\frac{m_{A}+m_{B}}{2}$, where | ***m*** | ≪ 1 because of the strong antiferromagnetic exchange coupling. Following the procedure given in ^[^ ^40^ ^]^ but now with the dual SOT included, it can be shown that *m* is expressed as a function of *n*: In a static state,

(4) $$m\;=\frac{1}{H_{\mathrm{IEC}}}\;n\times \left(-n\times H+H_{\mathrm{AIEC}}+\Delta H^{\mathrm{SOT}}\right)$$

where $\Delta \;H^{\mathrm{SOT}}=H_{A}^{\mathrm{SOT}}\;-H_{B}^{\mathrm{SOT}}$, and *H*_IEC_ and ***H***_AIEC_ are the effective fields associated with the IEC and AIEC, respectively. The first term in Equation (4) simply represents the field‐induced canting moment. Interestingly, as indicated by the second and third terms, the AIEC and the dual SOT play the equivalent role when it comes to their contribution to ***m***. As ***n*** ≈ ± ***z*** in each domain, the *y* component of ***H***_AIEC_ + Δ ***H*** ^SOT^ develops the *x* component of ***m*** in the domains, which directly couples to *H_x_* via the Zeeman interaction. The DW structure formed by ***m*** following that of ***n*** is therefore driven into motion by *H_x_*, accompanied by the entire DWM. The part due to Δ ***H*** ^SOT^ is the CIDWM due to the dual SOT, a mechanism which has never been discussed. We note that, while the part due to ***H***_AIEC_ has been long known in the context of bulk antiferromagnets,^[^ ^40^ ^]^ where ***H***_AIEC_ originates rather from the crystalline symmetry, it has never been demonstrated for a DW in a synthetic thin film system. If |**D**_AIEC_| becomes sufficiently large, the AIEC could affect *v*_dw_ via stabilizing a particular DW configuration (Néel, Bloch or intermediate) depending on *θ*_D_. While there are possible directions for further theoretical analysis, these are beyond the scope of the current work and have to be reserved for another study.

## 4 Conclusion

The roles of dual SOT and the AIEC for the CIDWM were experimentally and theoretically studied for the perpendicularly‐magnetized synthetic antiferromagnets using a Pt/Co/Ir/Co/Pt multilayer. The electric current application nucleates a reversed magnetic domain and shows the displacement of the domain wall existing between the head‐to‐head and tail‐to‐tail domains. From the electric current direction for CIDWM and magnetic field dependence of *v*_dw_, we can conclude that the dual SOT plays the major role for CIDWM. The AIEC field contributed to the reduction of the current density required for nucleating the revised domain and leads to an increase in *v*_dw_. These facts suggest that the existence of AIEC leads to the improvement of performance of CIDWM in these specially designed devices. To understand our results, we developed an appropriate theoretical model for CIDWM by dual SOT with AIEC. Our results provide a new avenue to design highly efficient SOT domain‐wall devices based on a synthetic antiferromagnet.

## 5 Experimental Section

### Thin Film Preparation and Device Fabrication

The multilayer stacks of Ta (2)/Pt (3)/Co (0.65 or *t*_Co_)/Ir (1.3)/Co (0.9)/Pt (3)/Ta (1) (thickness in nanometer) were deposited on a Si‐O substrate using dc magnetron sputtering at room temperature with an Ar pressure of 0.4 Pa. Before deposition, the chamber was evacuated to a base pressure below 6.0 × 10^−6^ Pa. The area of deposited films was 9 × 9 mm^2^, and *t*_Co_ was continuously varied in the length of 9 mm using a linear moving shutter. The thin films were patterned into a Hall‐bar‐shape using electron beam lithography and Ar‐ion milling. The channel width was 5 µm. The electrical contact pads of Cr (20 nm)/Au (200 nm) were fabricated using photolithography and ion‐beam sputtering.

### Transport Property Measurement

The transverse resistance *R_xy_* was detected by applying an ac charge current of 20 µA with a frequency of 9997 Hz using a lock‐in amplifier SR830. *R_xy_* as a function of out‐of‐plane magnetic field *H*_*z*_ was measured with and without the additional in‐plane magnetic field *H*_ip_ at room temperature.

### Magneto‐Optical Kerr Effect Imaging

The domain wall motion was visualized using a commercial Evico magnetics GmbH Kerr microscopy with an in‐plane magnetic field coil. A pulse‐shaped charge current with a pulse width of 500 ns was applied in a device using a function generator Agilent 33250A. The amplifier with a gain of 40 dB was connected between a function generator and a device. The pulse‐shaped signal was detected using an oscilloscope Tektronix OPO7354. The external magnetic fields in the range of from 30 to 100 mT were applied during the experiments.

## Conflict of Interest

The authors declare no conflict of interest.

## Supporting information



Supporting Information

## Data Availability

The data that support the findings of this study are available from the corresponding author upon reasonable request.
