# Supplementary material for: Efficient Manipulation of Magnetic Domain Wall by Dual Spin‐Orbit Torque in Synthetic Antiferromagnets
Source: Adv Sci (Weinh). 2025 Oct 17;12(48):e14598. doi: 10.1002/advs.202514598 (PMC12752659; doi:10.1002/advs.202514598)
Supplement: Supplementary file 1 — Supporting Information [file ADVS-12-e14598-s001.pdf]

Supporting Information

Efficient Manipulation of Magnetic Domain Wall by Dual Spin-Orbit Torque in Synthetic Antiferromagnets

*Hiroto Masuda, Yuta Yamane, Takaaki Dohi, Takumi Yamazaki, Rajkumar Modak, Ken-ichi Uchida, Jun'ichi Ieda, Mathias Kläui, Koki Takanashi, and Takeshi Seki**

Hiroto Masuda, Takumi Yamazaki, Koki Takanashi, and Takeshi Seki
Institute for Materials Research, Tohoku University, Sendai 980-8577, Japan
E-mail: takeshi.seki@tohoku.ac.jp

Yuta Yamane
Frontier Research Institute for Interdisciplinary Sciences, Tohoku University, Sendai 980-8578, Japan

Yuta Yamane, Takaaki Dohi
Research Institute of Electrical Communication, Tohoku University, Sendai 980-8577, Japan

Rajkumar Modak, Ken-ichi Uchida
Research Center for Magnetic and Spintronic Materials, National Institute for Materials Science, Tsukuba 305-0047, Japan

Rajkumar Modak, Ken-ichi Uchida
Department of Advanced Materials Science, Graduate School of Frontier Sciences, The University of Tokyo, Kashiwa 277-8561, Japan

Jun'ichi Ieda, Koki Takanashi
Advanced Science Research Center, Japan Atomic Energy Agency, Tokai 319-1195, Japan

Takaaki Dohi, Mathias Kläui
Institut für Physik, Johannes Gutenberg-Universität Mainz, Staudingerweg 7, 55128 Mainz, Germany

Takeshi Seki
Center for Science and Innovation in Spintronics, Tohoku University, Sendai 980-8577, Japan

Takeshi Seki
International Center for Synchrotron Radiation Innovation Smart, Tohoku University, Sendai 980-8577, Japan

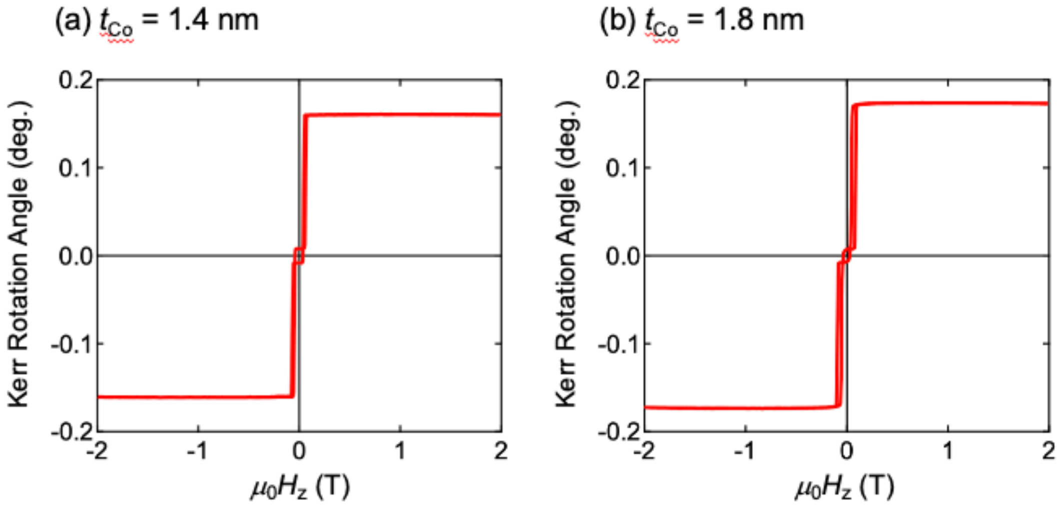

Figure S1. Polar magneto-optical Kerr effect (MOKE) loops at (a) t_{Co} = 1.4 nm and (b) 1.8 nm for the preliminary samples with the Co wedges of $0.6 \leq t_{Co} \leq 1.6$ nm ($\nabla t_{Co} = 1.1 \times 10^{-7}$) and $0.6 \leq t_{Co} \leq 2.1$ nm ($\nabla t_{Co} = 1.7 \times 10^{-7}$).

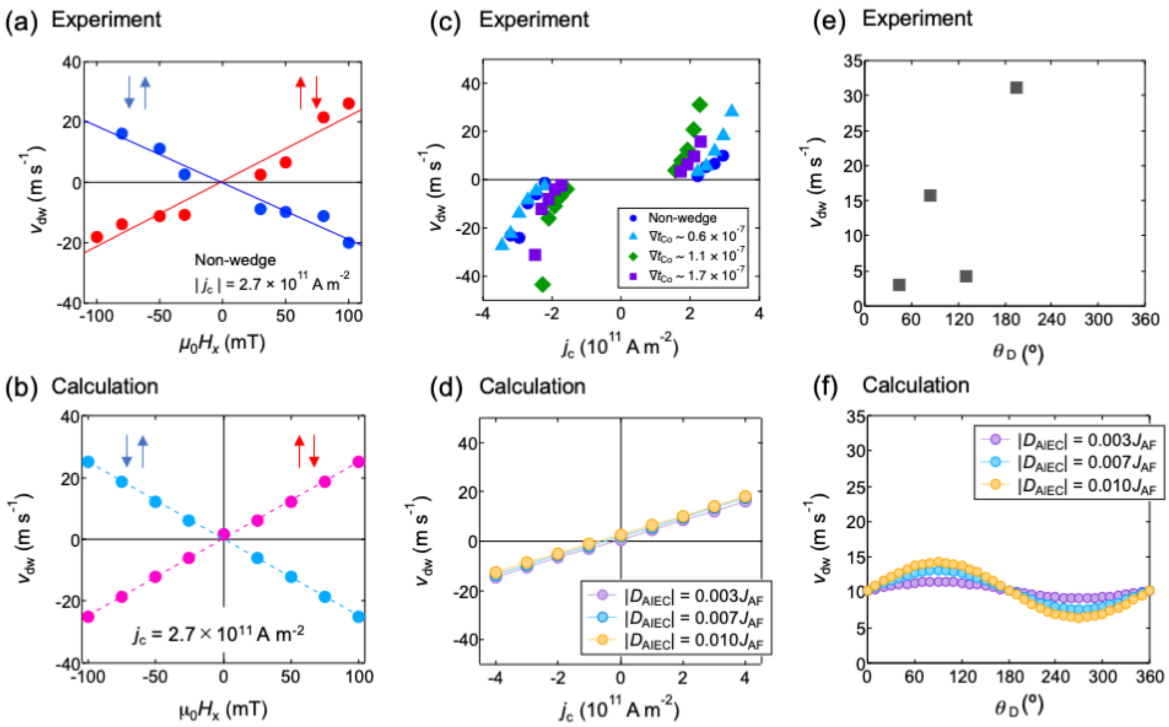

Figure S2. Comparison between experimental results and numerical simulations. (a) Experimental and (b) calculated v_{dw} as a function of H_x for the non-wedged device under the application of $j_c = 2.7 \times 10^{11}$ A m^{-2}, which are shown in Fig. 3c and Fig. 5a of main text, respectively. (c) Experimental and (d) calculated v_{dw} as a function of j_c under the application of $\mu_0 H_x = 50$ mT, which are shown in Fig. 3d and Fig. 5b of main text, respectively. (e) Experimental and (f) calculated v_{dw} as a function of θ_D under the application of $j_c = 2.3 \times 10^{11}$ A m^{-2} and $\mu_0 H_x = 50$ mT, which are shown in Fig. 5d and Fig. 5c of main text, respectively.
